# VEGFR-1 blockade disrupts peri-implantation decidual angiogenesis and macrophage recruitment

**DOI:** 10.1186/2045-824X-6-16

**Published:** 2014-08-01

**Authors:** Nataki C Douglas, Ralf C Zimmermann, Qian Kun Tan, Chantae S Sullivan-Pyke, Mark V Sauer, Jan K Kitajewski, Carrie J Shawber

**Affiliations:** 1 Department of Obstetrics and Gynecology, PH 16–64, Division of Reproductive Endocrinology and Infertility, Columbia University Medical Center, 622 W. 168th Street, New York, NY 10032, USA; 2 Department of Obstetrics and Gynecology, Division of Reproductive Sciences, College of Physicians and Surgeons, Columbia University Medical Center, 630 West 168th St, New York, NY 10032, USA

**Keywords:** VEGFR-1, Decidua, Uterus, Implantation, Endothelial cells, Macrophages, Angiogenesis

## Abstract

**Background:**

Angiogenesis and macrophage recruitment to the uterus are key features of uterine decidualization; the progesterone-mediated uterine changes that allow for embryo implantation and initiation of pregnancy. In the current study, we characterized the expression of vascular endothelial growth factor receptor-1 (VEGFR-1) in macrophages and endothelial cells of the peri-implantation uterus and determined if VEGFR-1 function is required for decidual angiogenesis, macrophage recruitment, and/or the establishment of pregnancy.

**Methods:**

Expression of VEGFR-1 in uterine endothelial cells and macrophages was determined with immunohistochemistry. To assess the effect of continuous VEGFR-1 blockade, adult female mice were given VEGFR-1 blocking antibody, MF-1, every 3 days for 18 days. After 6 doses, females were mated and a final dose of MF-1 was given on embryonic day 3.5. Endothelial cells and macrophages were quantified on embryonic day 7.5. Pregnancy was analyzed on embryonic days 7.5 and 10.5.

**Results:**

F4/80^+^ macrophages are observed throughout the stroma and are abundant adjacent to the endometrial lumen and glands prior to embryo implantation and scatter throughout the decidua post implantation. VEGFR-1 expression is restricted to the uterine endothelial cells. F4/80^+^ macrophages were often found adjacent to VEGFR-1^+^ endothelial cells in the primary decidual zone. Continuous VEGFR-1 blockade correlates with a significant reduction in decidual vascular and macrophage density, but does not affect embryo implantation or maintenance of pregnancy up to embryonic day 10.5.

**Conclusions:**

We found that VEGFR-1 functions in both decidual angiogenesis and macrophage recruitment to the implantation site during pregnancy. VEGFR-1 is expressed by endothelial cells, however blocking VEGFR-1 function in endothelial cells results in reduced macrophage recruitment to the uterus. VEGFR-1 blockade did not compromise the establishment and/or maintenance of pregnancy.

## Introduction

Implantation of the fertilized oocyte induces uterine decidualization, the rapid proliferation and differentiation of stromal fibroblasts into glycogen and lipid rich decidual cells. Endothelial cells within the decidua proliferate to form a rudimentary vascular plexus necessary to maintain pregnancy prior to placenta development [1-3]. Cells of the innate immune system, including natural killer cells and macrophages, infiltrate the decidua and function to establish and maintain maternal–fetal tolerance [4,5]. These events are coordinated by ovarian estrogen and progesterone. Angiogenesis during decidualization is required for a successful pregnancy, however the signaling pathways that regulate this process have yet to be fully characterized.

In mice and non-human primates, VEGF mediates the increased uterine vascular permeability and decidual angiogenesis required for embryo implantation [6-8]. We have shown that the VEGF receptors (VEGFR-1, VEGFR-2, and VEGFR-3) are expressed in distinct patterns in the peri-implantation uterine decidua [9]. To investigate the requirement for VEGF receptor signaling in the decidua, we administered a single, peri-implantation (E3.75) dose of blocking antibody against VEGFR-1, VEGFR-2, or VEGFR-3, in a progesterone-replaced, ovariectomized mouse model. Inhibition of VEGF-A receptor, VEGFR-2, blocked decidual angiogenesis observed at E7.5 and resulted in an aborted pregnancy prior to E10.5, while VEGFR-3 inhibition moderately reduced decidual angiogenesis but had no effect on pregnancy [9]. In contrast, VEGFR-1 blockade at a single E3.75 time-point did not effect decidual angiogenesis or disrupt pregnancy [9].

Embryo implantation and trophoblast invasion create a pro-inflammatory environment leading to the recruitment of immune cells that mediate maternal tolerance to the semi-allogeneic embryo [5,10]. Decidual macrophages are the second most abundant immune cell population at the implantation site, comprising 20-30% of immune cells in the uterine decidua [4,11,12]. Dysregulated macrophage activation in the decidua has been implicated in recurrent miscarriages [13]. Decidual macrophage numbers, as well as the proportion of pro-apoptotic Fas ligand expressing decidual macrophages, are increased in spontaneous miscarriages [14]. Thus, understanding the signaling pathways that regulate decidual macrophages may elucidate causes of early pregnancy failures.

Macrophages are closely associated with endothelial cells during physiologic angiogenesis during ovarian corpus luteum development and postnatal retinal development, [15,16]. Activation of macrophage VEGFR-1 has a role in macrophage recruitment to sites of active angiogenesis [17,18]. Macrophages have been implicated in promoting angiogenesis by releasing pro-angiogenic factors such as VEGF-A and angiopoietin [15] and in anastomosis, the formation of a bridge between two angiogenic tip cells followed by vascular sprout fusion [15,19]. Ablation of ovarian macrophages leads to significant endothelial cell depletion and hemorrhage [16,20]. Macrophages have been implicated in the pregnant uterus, including acting in the coordination of the maternal immune response and promoting angiogenesis. However, the relationship between decidual macrophages, endothelial cells and VEGFR-1 has not been investigated.

VEGFR-1 binds VEGF-A, VEGF-B and placental growth factor (PlGF) [21]. *VEGFR-1* gene encodes two mRNA splice variants. The full-length VEGFR-1 variant encodes a membrane bound receptor with classic intracellular tyrosine kinase signaling that positively regulates angiogenesis. The alternatively spliced variant encodes the VEGFR-1 extracellular domain resulting in a secreted protein known as soluble VEGFR-1 (sFlt-1) that is anti-angiogenic [22,23]. sFlt-1 sequesters the VEGFR-2 ligand, VEGF-A as well as, the VEGFR-1 specific ligands, VEGF-B and PlGF. Thus, sFlt-1 functions as a negative regulator of both VEGFR-2 and full-length VEGFR-1 signaling and is necessary for proper embryonic angiogenesis [23,24]. VEGFR-1 null (*Flt-1*^*-/-*^) mutant embryos die because of an overgrowth of vascular endothelial cells [24]. Mice with deletion of the VEGFR-1 Tyrosine Kinase (TK) domain (*Flt-1 TK*^*-/-*^) are viable and have normal blood vessel development, but have altered macrophage migration [25]. In pathologic conditions, such as cancer and inflammatory diseases, full length VEGFR-1 signaling promotes angiogenesis and activates/mediates migration of macrophage-lineage cells [26]. Thus, the context and abundance of sFlt-1 and full-length VEGFR-1 determine the pro- or anti-angiogenic effect of VEGFR-1.

In this study, we determined the expression pattern of VEGFR-1 with respect to endothelial cells and macrophages in the pre- and post-implantation uterus. VEGFR-1 neutralizing antibodies were administered prior to and during implantation. Our goal was to determine if inhibition of VEGFR-1 affects decidual angiogenesis, macrophage recruitment, and/or the establishment and maintenance of pregnancy in mice. Using two different VEGFR-1 blocking antibodies, MF-1 and R&D Systems AF471 that prevent ligand binding to VEGFR-1, we found that both vascular density and macrophage recruitment to the pregnant uterus were significantly reduced. Despite this, embryos implanted in the uterus and pregnancy progressed after VEGFR-1 blockade. We conclude that VEGFR-1 activity is required for proper decidual angiogenesis and macrophage recruitment to the implantation site during pregnancy.

## Materials and methods

### Animal model

The Columbia University Institutional Animal Care and Use Committee approved animal studies. Adult wild-type CD1 female mice and CD1 male mice of proven fertility were used. When mice were bred, noon on the day a mating plug was observed was designated embryonic day (E) 0.5. Uteri of non-pregnant female mice and pregnant females at E3.5 and E6.5 were embedded in Tissue-Tek® O.C.T.™ Compound (Sakura Fine Technical Co, Ltd, Tokyo, Japan), snap-frozen on dry ice in ethanol and stored at -80°C.

To determine the effect of continuous VEGFR-1 blockade, females were intraperitoneally (i.p.) injected with VEGFR-1 blocking monoclonal antibody (MF-1, 132 mg/kg animal, ImClone Systems, Inc.; n = 5 mice), anti-mouse VEGFR-1 antibody (AF471, 2 mg/kg animal, R&D systems; n = 1 mice), or saline (n = 5 mice) every 3 days for 18 days. MF-1 has an elimination half-life of 3 days and reaches maximal plasma concentrations 6 hours after dosing [27]. After 6 doses, females were mated and a final dose of antibody or saline was given on E3.5. Pregnant females were sacrificed on E7.5. Implantation sites were counted, uterine weight measured, and uteri freshly frozen in O.C.T.™ compound for sectioning. Pregnant females were sacrificed on E10.5 to determine the number of implantation sites and uterine weight.

### Histological staining

For uterine analyses, 5 μm transverse sections through the non-pregnant and E3.5 uteri were generated. For implantation site analyses, frontal sections through uterus/implantation site were generated for E6.5 at 7 μm and for E7.5 with VEGFR-1 blockade at 12 μm. Implantation was confirmed by H&E staining every 5th section. Specific staining was performed at least 3 times and 5 different uterine sections or implantation sites were analyzed.

Sections were stained as previously described [27]. Primary antibodies included anti-mouse VEGFR-1 (R&D Systems, AF471), anti-mouse F4/80 (eBioscience, 14–4801), anti-mouse CD31 (BD Biosciences, 553370), anti-mouse endomucin (Santa Cruz, sc-65495), anti-mouse VE-cadherin (BD Biosciences, 550548), and anti-mouse CD11b (Abcam ab8878). For IHC staining, biotin rabbit anti-goat IgG (Vector, BA-5000), biotin goat anti-rat IgG (BD Biosciences, 559286), the avidin/biotin blocking kit (Vector, SP-2001), the Vectastain ABC kit and DAB substrate kit (Vector, SK-4100) were used. Sections were counterstained with hematoxylin. For IF experiments, the following secondary antibodies were used: donkey anti goat-IgG Alexa-Fluor 594 (Invitrogen, A11058) and donkey anti rat-IgG Alexa-Fluor 488 (Invitrogen, A2108). Slides were covered with Vectashield containing with 4′, 6-diamidino-2-phenylindole (Vector, H-1200) for nuclear visualization.

### Imaging

IHC and H&E staining images were captured with a Nikon Eclipse E800 microscope and Nikon DXM 1200 digital camera and NIS-Elements D3.10 software or ImagePro Plus v.4.01 software. Fluorescent images were captured using a Nikon A1 scanning confocal microscope on an Eclipse Ti microscope stand (Nikon Instruments, Melville, NY). Standard lasers and filters were used to image DAPI, AlexaFluor 488, and TRITC. Images were taken using the 10×/0.4 and 60×/1.49 objectives. Representative Z-stacked maximum intensity images are shown.

### Quantitation of decidual macrophage and vascular density

The percentage of decidua occupied by blood vessels or macrophages was calculated from E7.5 decidua frontal uterine sections by dividing the total area of CD31 or F4/80 staining by the area of the decidua multiplied by 100. Images were captured using a Nikon Eclipse E800 microscope and Nikon DXM 1200 digital camera, and data were processed using ImagePro Plus Version 4.01 (Media Cybernetics) [28].

### Serum progesterone levels

Blood was obtained from all animals by cardiopuncture. Serum progesterone levels were measured using a competitive chemiluminescent immunoassay (Diagnostic Products Corp./Siemens) [9].

### Statistical analysis

Data are presented as mean ± standard error of the mean (sem). Unpaired Student t-test was used to compare sample means. *P* < 0.05 was considered a statistically significant difference. Statistical analyses were performed using the Statistical Package for Social Science version 15.0 (SPSS, Inc., Chicago, Il).

## Results

### Endothelial and macrophage expression of VEGFR-1 in the peri-implantation uterus

**Figure 1:**
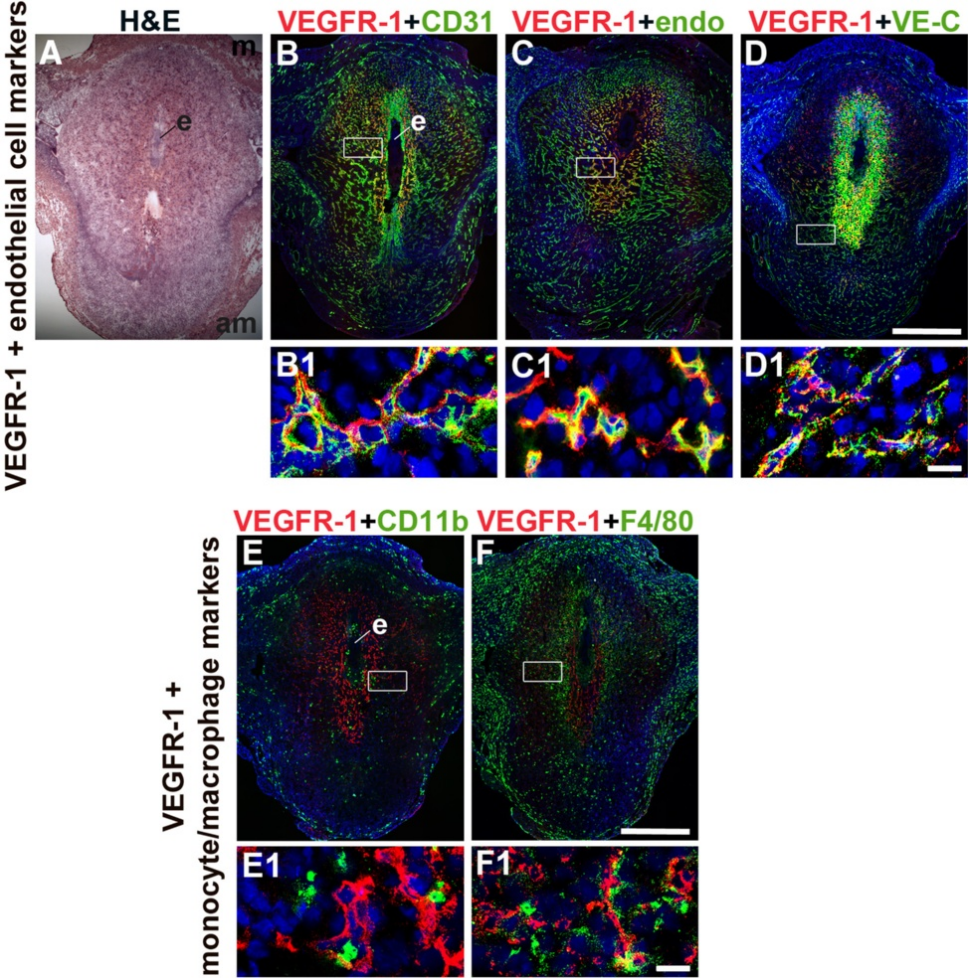
**VEGFR-1 expression in endothelial cells and macrophages in the post-implantation uterus.** H&E and double staining IF were performed on E6.5 frontal uterine sections. **(A)** H&E of a post-implantation mouse uterus showing the embryo (e), anti-mesometrial (am) and mesometrial (m) areas. **(B-F)** VEGFR-1^+^ cells (red) are observed in the decidua, with abundant expression in the primary decidual zone surrounding the implanted embryo. **(B)** Double-staining for VEGFR-1^+^ (red) and CD31^+^ (green) cells demonstrates expression of VEGFR-1 in a subset of CD31^+^ ECs. **(C)** Double-staining for VEGFR-1^+^ (red) and endomucin^+^ (green) cells demonstrates expression of VEGFR-1 in a subset of endomucin^+^ ECs. **(D)** Double-staining for VEGFR-1^+^ (red) and VE-cadherin^+^ (green) cells demonstrates expression of VEGFR-1 in VE-cadherin^+^ ECs. **(E)** Double-staining for VEGFR-1^+^ (red) and CD11b^+^ (green) cells demonstrates that VEGFR-1 is not expressed in CD11b^+^ monocytes. **(F)** Double-staining for VEGFR-1^+^ (red) and F4/80^+^ (green) cells demonstrates that VEGFR-1 is not expressed in F4/80^+^ macrophages. VEGFR-1^+^ cells are adjacent to CD11b^+^ monocytes and F4/80^+^ macrophages. White boxes in **(B-F)** indicate areas of the uteri magnified below **(B1-F1)**. **(A-F)** Scale bar = 500 μm. **(B1-F1)** Scale bar = 20 μm.

We determined the expression of VEGFR-1 in uterine endothelial cells (ECs) and macrophages in the post-implantation murine decidua at E6.5 (Figure 1). To determine EC expression, VEGFR-1 was co-stained with three endothelial cell markers, CD31 [9,28,29], endomucin [30] and VE-cadherin [31,32]. To determine macrophage VEGFR-1 expression, uterine sections were stained for VEGFR-1 and either the monocyte marker, CD11b [33,34] or the macrophage marker, F4/80 [35-37].

At E6.5, the embryo is readily detected in the post-implantation uterus (Figure 1A). VEGFR-1^+^ cells are most abundant in the decidua directly surrounding the implanted embryo or the primary decidual zone (Figure 1B–F). CD31^+^ and endomucin^+^ ECs are observed throughout the decidua (Figure 1B, C), while VE-cadherin^+^ ECs are most abundant in the anti-mesometrial decidua (Figure 1D). The positive VE-cadherin staining directly adjacent to the implanted embryo does not have a vascular pattern and does not represent ECs (Figure 1D). VEGFR-1 is expressed in decidual ECs that express CD31 (Figure 1B), endomucin (Figure 1C) or VE-cadherin (Figure 1D). CD11b^+^ monocytes and F4/80^+^ macrophages are scattered throughout the decidua, but their distribution patterns differ (Figure 1E, F). VEGFR-1 expression is not detected in CD11b^+^ or F4/80^+^ decidual monocytes/macrophages (Figure 1E, F), in contrast to VEGFR-1 expression in decidual ECs. VEGFR-1 is expressed in cells that are directly adjacent to decidual monocytes/macrophages. Since VEGFR-1 expression closely overlaps with CD31 expression, CD31 was used as the EC marker for all additional experiments.

### VEGFR-1 is expressed in endothelial cells of the non-pregnant uterus

**Figure 2:**
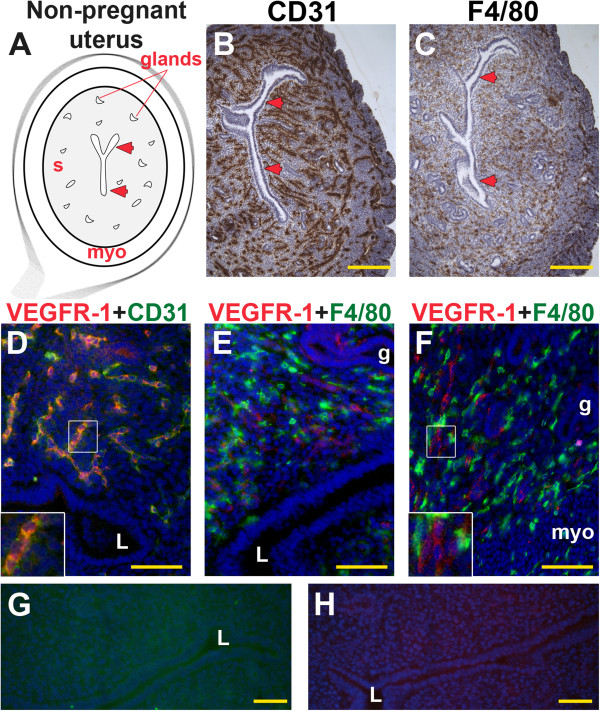
**VEGFR-1, CD31 and F4/80 expression in the non-pregnant uterus.** IHC and double staining IF were performed on non-pregnant uterine cross-sections. **(A)** Schematic representation of a non-pregnant mouse uterus showing lumen (arrowheads), endometrial glands, stroma (s), and myometrium (myo). **(B)** ECs, detected by CD31 staining (brown), are observed throughout the stroma and myometrium. **(C)** Macrophages, detected by F4/80 staining (brown), are observed throughout the stroma and myometrium. **(D)** Double-staining for VEGFR-1^+^ (red) and CD31^+^ (green) demonstrates expression of VEGFR-1 in a subset of CD31^+^ ECs throughout the stroma. **(E, F)** VEGFR-1^+^ cells (red) and F4/80^+^ macrophages (green) are distributed throughout the stroma. VEGFR-1 and F4/80 co-expression is not observed. The inset **(F)** highlights contact of adjacent VEGFR-1^+^ cells and F4/80^+^ macrophages. **(G, H)** IF controls with secondary antibodies alone show no specific staining. L, lumen; g, endometrial gland. **(B, C)** Scale bar = 100 μm. **(D-H)** Scale bar = 50 μm.

In the non-pregnant uterus, CD31^+^ ECs and F4/80^+^ macrophages are distributed throughout the surrounding stroma and myometrium, but excluded from the uterine lumen and tubular endometrial glands that are lined by a single layer of columnar epithelium (Figure 2A–C). VEGFR-1 expression is restricted to a subset of stromal ECs (Figure 2D). VEGFR-1 is not expressed by F4/80^+^ macrophages, but is expressed on cells adjacent to stromal macrophages (Figure 2E, F).

### VEGFR-1 is expressed in endothelial cells of the pre-implantation uterus

**Figure 3:**
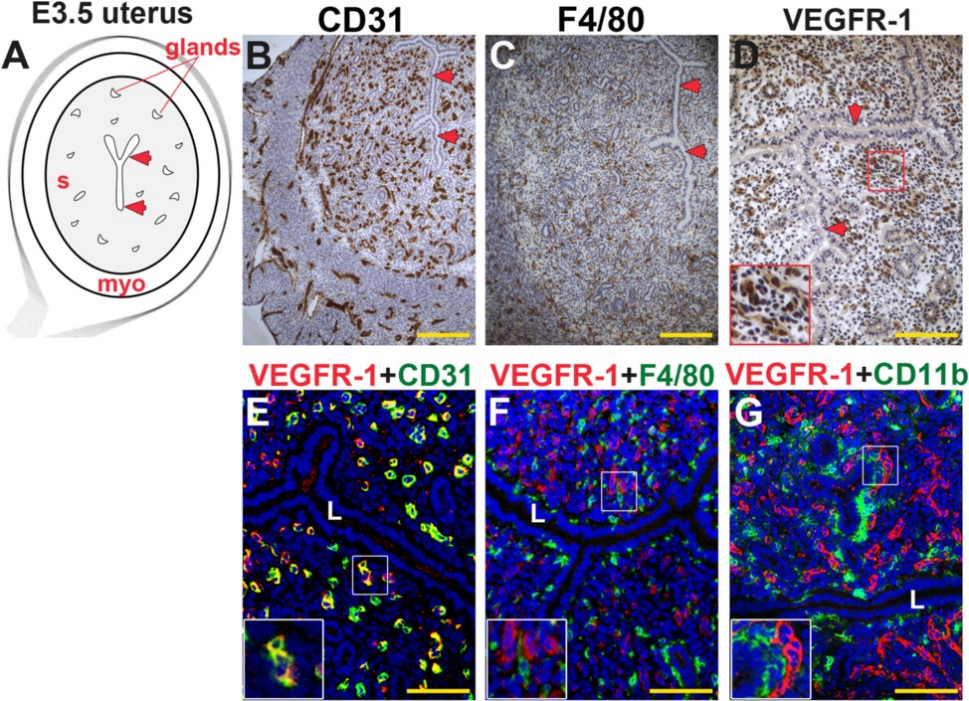
**VEGFR-1, CD31, F4/80, and CD11b expression in the pre-implantation mouse uterus.** IHC and double staining IF were performed on E3.5 uterine cross-sections. **(A)** Schematic representation of an E3.5 mouse uterus showing lumen (arrowheads), glands, stroma (s), and myometrium (myo). **(B)** ECs, detected by CD31 staining (brown), are observed throughout the stroma and myometrium, similar to the non-pregnant state. **(C)** Macrophages, detected by F4/80 staining (brown), are observed throughout the stroma and are abundant adjacent to the lumen and glands at E3.5. **(D)** VEGFR-1^+^ cells (brown), are distributed throughout the stroma and cell associated VEGFR-1 expression highlighted in the inset. **(E)** Double staining for VEGFR-1 (red) and CD31 (green) demonstrates expression of VEGFR-1 on CD31^+^ ECs throughout the stroma. **(F)** VEGFR-1^+^ cells (red) and F4/80^+^ macrophages (green) are distributed throughout the stroma. VEGFR-1 and F4/80 co-expression is not observed. **(G)** VEGFR-1^+^ cells (red) and CD11b^+^ monocytes (green) are distributed throughout the stroma. VEGFR-1 and CD11b co-expression is not observed. L, lumen. Scale bars B, C = 100 μm. Scale bars D – F = 50 μm.

By E3.5, progesterone secretion by ovarian corpora lutea has been initiated to prepare the endometrium for embryo implantation. The gross appearance of the E3.5 pre-implantation uterus is similar to the non-pregnant uterus (Figures 2A and 3A). As in the non-pregnant state, CD31^+^ ECs (Figure 3B) and F4/80^+^ macrophages (Figure 3C) are distributed throughout the stroma and myometrium. Similar to the non-pregnant state, CD31^+^ ECs and F4/80^+^ macrophages (Figure 3C, F) are abundant adjacent to the lumen and glands at E3.5. VEGFR-1^+^ cells are observed throughout the stroma (Figure 3D). VEGFR-1 expression was cell associated consistent with the antibody detecting full-length VEGFR-1 on the cell surface (Figure 3E, inset). Co-staining with CD31 reveals that most VEGFR-1^+^ cells are ECs (Figure 3E). As in the non-pregnant state, VEGFR-1^+^ cells are found adjacent to F4/80^+^ macrophages (Figure 3F) and CD11b^+^ monocytes (Figure 3G).

### VEGFR-1 is expressed in endothelial cells of the post-implantation uterus

**Figure 4:**
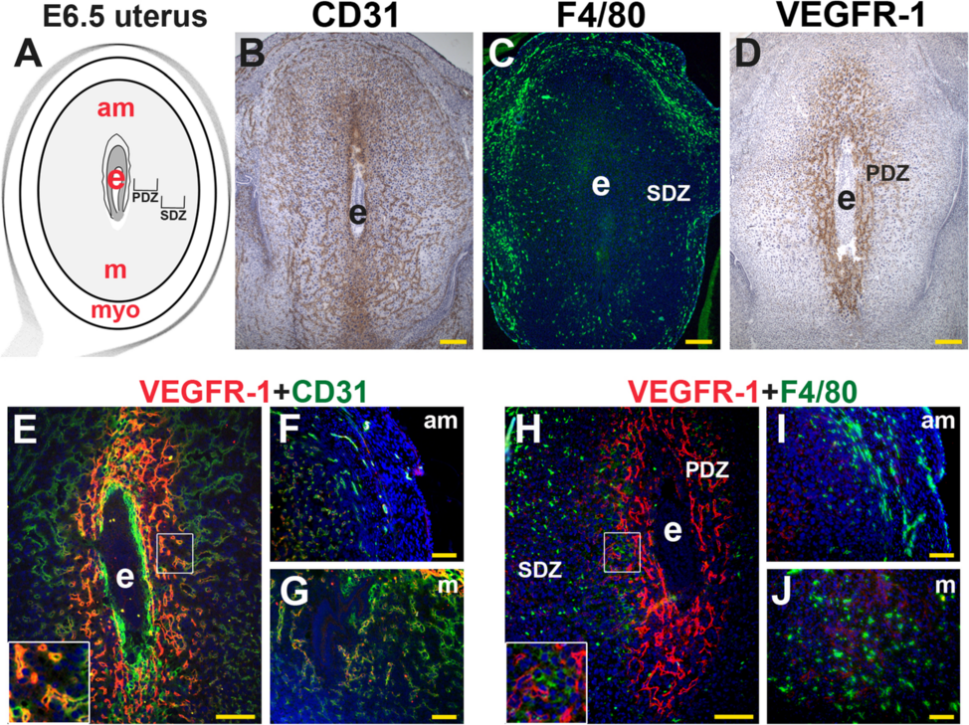
**VEGFR-1, CD31, and F4/80 in the post-implantation mouse uterus.** IHC and double staining IF were performed on E6.5 frontal uterine sections. **(A)** Schematic representation of an E6.5 mouse uterus showing the embryo (e), anti-mesometrial (am) and mesometrial (m) areas, and myometrium (myo). **(B)** CD31^+^ ECs (brown) are abundant in the decidua around the implanted embryo and the myometrium. **(C)** A majority of F4/80^+^ macrophages (green) are observed in the secondary decidual zone (SDZ) and myometrium. **(D)** A majority of VEGFR-1^+^ cells (brown) are observed in the primary decidual zone (PDZ) directly adjacent to the implanted embryo. **(E-F)** Double staining of VEGFR-1 (red) and CD31^+^ (green) demonstrates **(E)** co-expression in the ECs within the PDZ (inset), **(F, G)** and minimal co-expression of VEGFR-1 and CD31 in the abundant ECs in the SDZ of the anti-mesometrial and mesometrial poles. **(H)** Double staining for VEGFR-1 (red) and F4/80^+^ (green) shows VEGFR-1^+^ cells adjacent to F4/80^+^ macrophages in the PDZ (inset). **(I, J)** F4/80^+^ macrophages are abundant in the SDZ of the anti-mesometrial pole and mesometrial poles, where VEGFR-1 expression is low to absent. **(B-D, H)** Scale bars = 100 μm. **(F, G, I, J)** Scale bars = 50 μm.

With embryo-uterine contact during implantation, stromal cells are transformed into large, polygonal, glycogen and lipid rich decidual cells. By E6.5, the primary and secondary decidual zones are defined by morphologic differences in decidual cells, as well as differential expression of tight junction proteins and VEGF receptors [9,29,38,39] (Figure 4A). CD31^+^ ECs are abundant throughout the decidua, the endometrium surrounding the implanted embryo, and myometrium (Figure 4B, F, and G). F4/80^+^ macrophages are scattered throughout the decidua and most abundant in the myometrium and stroma directly adjacent to the myometrium (Figure 4C, I, J). Within the decidua, the secondary decidual zone (SDZ) contains the highest concentration of F4/80^+^ macrophages (Figure 4C). VEGFR-1^+^ cells are most abundant in the primary decidual zone (PDZ) (Figure 4D, E, H). Most VEGFR-1^+^ cells are ECs (Figure 4E) and within the PDZ, VEGFR-1^+^ cells are found adjacent to F4/80^+^ macrophages (Figure 4H). As in the pre-implantation uterus, VEGFR-1 expression in decidual ECs is consistent with full-length VEGFR-1 (Figure 4E, H, insets). The distribution pattern of VEGFR-1^+^ ECs and macrophages suggests that VEGFR-1^+^ EC and F4/80^+^ macrophages are in direct contact in both the non-pregnant, peri-implantation and post-implantation uterus.

### Effects of VEGFR-1 blockade on pregnancy, decidual macrophages and vascular density

**Figure 5:**
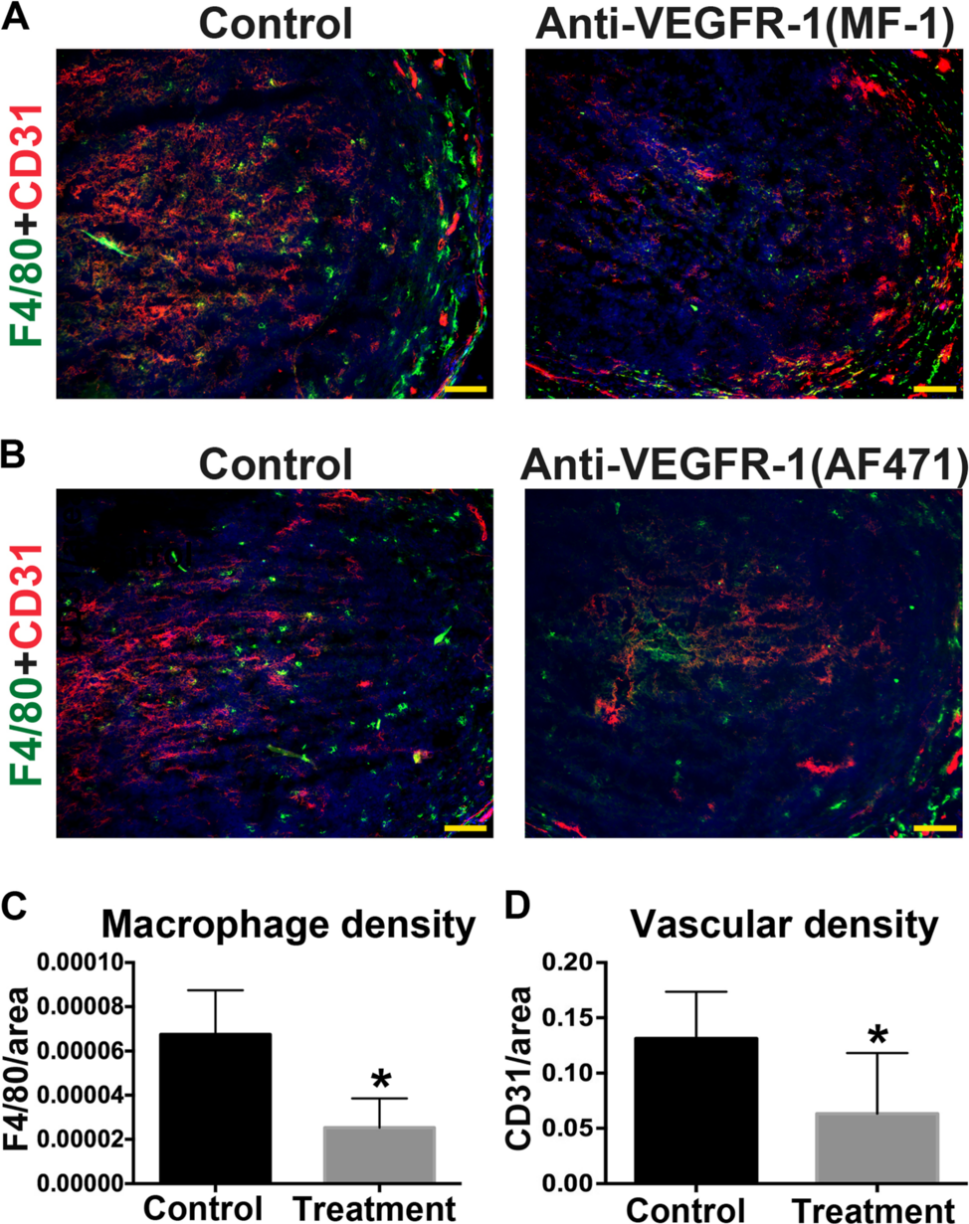
**VEGFR-1 blockade reduces peri-implantation macrophage and vascular density at E7.5. (A, B)** Double staining for F4/80 (green) and CD31 (red) in E7.5 uterine frontal sections from mice treated with VEGFR-1 blocking antibodies (MF-1 or AF471) or from control mice. Reduced expression of F4/80 and CD31 is observed in treatment groups. Scale bars = 50 μm. **(C, D)** F4/80^+^ or CD31^+^ signal was determined and normalized by total decidual area. F4/80^+^ macrophage and CD31^+^ EC density is significantly reduced in uteri of mice that received MF-1 compared to controls.

Previous studies demonstrated that a single dose of VEGFR-1 blocking antibody MF-1, administered on E3.75, had no effect on decidual angiogenesis or pregnancy [9]. In this study, we administered 6 doses of MF-1 prior to mating and a single dose on E3.5, one day prior to implantation, to determine the effect of continuous VEGFR-1 blockade on pregnancy. Administration of VEGFR-1 blocking antibody MF-1 had no effect on the number of implantation sites or uterine weights at E7.5 and E10.5 (Table 1). Histological analysis of uterine tissues sections confirmed normal embryonic development at E7.5 and E10.5 (data not shown). However, differences in macrophage and vascular density were observed. At E7.5, F4/80^+^ macrophage and CD31^+^ EC densities were both significantly decreased in MF-1 treated uteri compared to controls (Figure 5 and Table 1). VEGFR-1 blockade with R&D Systems AF471 antibody also resulted in decreased macrophage numbers and vascular density at E7.5 (Figure 5B).

**Table 1:** Effect of anti-VEGFR-1 blocking antibody MF-1 on pregnancy, decidual macrophages and vascular density

	**Anti-VEGFR-1 (MF-1)**		**Control**	
	**E7.5**	**E10.5**	**E7.5**	**E10.5**
Implantation sites (#)	13 ± 0.6	12.1 ± 0.4	13 ± 0.3	11.8 ± 0.9
Uterine weights (g)	0.35 ± 0.01	2.1 ± 0.1	0.36 ± 0.02	2.3 ± 0.1
Serum progesterone (ng/mL)	23.4 ± 2.3		27.9 ± 1.1	
Macrophage density	6.7 × 10^-5^ ± 1.1 × 10^-6a^		2.5 × 10^-5^ ± 4.3 × 10^-7a^	
Vascular density	13.1 ± 0.2^b^		6.3 ± 0.2^b^	

^a, b^*P* < 0.01.

To confirm that blocking VEGFR-1 did not affect ovarian function, we measured serum progesterone levels at E7.5. The mean serum progesterone level was similar in MF-1 treated mice and controls (23.4 ± 2.3 vs. 27.9 ± 1.1 ng/mL, Table 1), indicating that VEGFR-1 blockage did not affect corpora lutea function in this model. Thus, the observed uterine defects can be attributed to the direct affects of VEGFR-1 blockade on the uterus.

## Discussion

We have previously shown that peri-implantation inhibition of VEGFR-2 significantly reduces vascular density and causes pregnancy loss by E10.5. We showed that only a subset of endothelial cells express VEGFR-1 and peri-implantation inhibition with a single dose of VEGFR-1 blocking antibody at E3.75 does not affect vascular density or pregnancy [9]. Our present evaluation of VEGFR-1 expression with respect to endothelial cells and macrophages shows that VEGFR-1 expression is mainly restricted to uterine and decidual endothelial cells, while VEGFR-1 is not expressed in monocytes and macrophages. Endothelial cells expressing VEGFR-1 were often in direct contact with CD11b^+^ monocytes and F4/80^+^ macrophages. Finally repeated pre-implantation injections of a VEGFR-1 blocking antibody, significantly decrease both the number of macrophages and vascular density in the decidua of E7.5 pregnant mice. Taken together, our results suggest that the decreased vascularity observed at E7.5 is a direct result of VEGFR-1 blockade, whereas the reduction in macrophages may be an indirect result of the decrease in VEGFR-1 function in the endothelial cells.

Treatment with MF-1, which blocks binding of VEGF-A and PlGF to VEGFR-1, can have pro- or anti-angiogenic effects, depending on the context of VEGFR-1 function [26,40-42]. PlGF signaling, via membrane-localization of full length VEGFR-1, plays a pro-angiogenic role in pathological angiogenesis [26,42]. In murine decidual angiogenesis, VEGFR-1 appears to be membrane associated consistent with full-length receptor expression (Figures 1 and 3E, H). Thus, the reduced angiogenesis we observed is likely due to blocking ligand-dependent VEGFR-1 pro-angiogenic signaling. However, it has been previously shown that the VEGFR-1 TK domain is not necessary for murine decidual angiogenesis [29]. Thus the pro-angiogenic role for VEGFR-1, we observed may be independent of VEGFR-1 TK signaling pathway.

We have shown that a single dose of MF-1 administered within 24 hours of implantation does not affect vascular density [9], but continuous VEGFR-1 blockade results in a 48% reduction in vascular density (Figure 5D). A greater than 50% reduction in decidual vascular density with VEGFR-2 blockade results in pregnancy loss, likely due to loss of angiogenesis in both the primary and secondary decidual zones [9]. In contrast, inhibition of VEGFR-3 results in a less than 50% decrease in primary decidual zone angiogenesis, but does not lead to pregnancy loss [9]. Combined, these data suggest that implantation and early pregnancy can tolerate a less than 50% reduction of vascular density in the primary decidual zone.

Macrophages are prominent in the adult ovary and uterus, two organs with physiological cyclical angiogenesis regulated by the VEGF/VEGFR-2 signaling pathway [9,28,43]. The number and phenotype of macrophages in the non-pregnant uterus changes with the ovarian cycle and additional macrophages are recruited into the endometrium during the peri-implantation period [20,44]. It has been shown that pre-implantation macrophage ablation prevents embryo implantation and results in infertility [20]. However, fertility is restored when macrophage-depleted mice are supplemented with exogenous progesterone suggesting a defect in corpora lutea function. In fact, macrophages have been shown to regulate corpus lutea development and function, specifically the synthesis of progesterone that is required for embryo implantation and establishing pregnancy [20,45]. Thus, uterine macrophages may not be necessary for embryo implantation. We found that pregnancy can tolerate a reduction of macrophages by 37% of normal. Taken together, the data suggest that macrophages do not have an obligatory role in the uterine decidualization and implantation of the embryo. However the role of macrophages after embryo implantation still remains to be elucidated.

## Competing interests

The authors declare that they have no competing interests.

## Authors’ contributions

ND prepared the manuscript and all figures. RZ and QKT did the experiments with MF-1 and R&D Systems AF471. CSP did the immunohistochemistry. JK and MS reviewed and edited the final manuscript. CS prepared and edited the manuscript. All authors read and approved the final manuscript.

## References

[B1] Dey SK Lim H Das SK Reese J Paria BC Daikoku T Wang H Molecular cues to implantation Endocr Rev 2004 25 341 373 15180948 10.1210/er.2003-0020

[B2] Cha J Sun X Dey SK Mechanisms of implantation: strategies for successful pregnancy Nat Med 2012 18 1754 1767 23223073 10.1038/nm.3012 PMC6322836

[B3] Wang H Dey SK Roadmap to embryo implantation: clues from mouse models Nat Rev Genet 2006 7 185 199 16485018 10.1038/nrg1808

[B4] Kabawat SE Mostoufi-Zadeh M Driscoll SG Bhan AK Implantation site in normal pregnancy. A study with monoclonal antibodies Am J Pathol 1985 118 76 84 3155596 PMC1887848

[B5] Fest S Aldo PB Abrahams VM Visintin I Alvero A Chen R Chavez SL Romero R Mor G Trophoblast-macrophage interactions: a regulatory network for the protection of pregnancy Am J Reprod Immunol 2007 57 55 66 17156192 10.1111/j.1600-0897.2006.00446.x

[B6] Rabbani ML Rogers PA Role of vascular endothelial growth factor in endometrial vascular events before implantation in rats Reproduction 2001 122 85 90 11425332 10.1530/rep.0.1220085

[B7] Rockwell LC Pillai S Olson CE Koos RD Inhibition of vascular endothelial growth factor/vascular permeability factor action blocks estrogen-induced uterine edema and implantation in rodents Biol Reprod 2002 67 1804 1810 12444056 10.1095/biolreprod.102.006700

[B8] Sengupta J Lalitkumar PG Najwa AR Charnock-Jones DS Evans AL Sharkey AM Smith SK Ghosh D Immunoneutralization of vascular endothelial growth factor inhibits pregnancy establishment in the rhesus monkey (Macaca mulatta) Reproduction 2007 133 1199 1211 17636174 10.1530/rep.1.01228

[B9] Douglas NC Tang H Gomez R Pytowski B Hicklin DJ Sauer CM Kitajewski J Sauer MV Zimmermann RC Vascular endothelial growth factor receptor 2 (VEGFR-2) functions to promote uterine decidual angiogenesis during early pregnancy in the mouse Endocrinology 2009 150 3845 3854 19406950 10.1210/en.2008-1207 PMC2717882

[B10] Co EC Gormley M Kapidzic M Rosen DB Scott MA Stolp HA McMaster M Lanier LL Barcena A Fisher SJ Maternal decidual macrophages inhibit NK cell killing of invasive cytotrophoblasts during human pregnancy Biol Reprod 2013 88 155 23553431 10.1095/biolreprod.112.099465 PMC4070869

[B11] Vince GS Starkey PM Jackson MC Sargent IL Redman CW Flow cytometric characterisation of cell populations in human pregnancy decidua and isolation of decidual macrophages J Immunol Methods 1990 132 181 189 2145368 10.1016/0022-1759(90)90028-t

[B12] Lessin DL Hunt JS King CR Wood GW Antigen expression by cells near the maternal-fetal interface Am J Reprod Immunol Microbiol 1988 16 1 7 3369615 10.1111/j.1600-0897.1988.tb00169.x

[B13] Wang WJ Hao CF Lin QD Dysregulation of macrophage activation by decidual regulatory T cells in unexplained recurrent miscarriage patients J Reprod Immunol 2011 92 97 102 22015003 10.1016/j.jri.2011.08.004

[B14] Guenther S Vrekoussis T Heublein S Bayer B Anz D Knabl J Navrozoglou I Dian D Friese K Makrigiannakis A Jeschke U Decidual macrophages Are significantly increased in spontaneous miscarriages and over-express FasL: a potential role for macrophages in trophoblast apoptosis Int J Mol Sci 2012 13 9069 9080 22942752 10.3390/ijms13079069 PMC3430283

[B15] Kofler NM Shawber CJ Kangsamaksin T Reed HO Galatioto J Kitajewski J Notch signaling in developmental and tumor angiogenesis Genes Cancer 2011 2 1106 1116 22866202 10.1177/1947601911423030 PMC3411124

[B16] Turner EC Hughes J Wilson H Clay M Mylonas KJ Kipari T Duncan WC Fraser HM Conditional ablation of macrophages disrupts ovarian vasculature Reproduction 2011 141 821 831 21393340 10.1530/REP-10-0327 PMC3101494

[B17] Pipp F Heil M Issbrucker K Ziegelhoeffer T Martin S van den Heuvel J Weich H Fernandez B Golomb G Carmeliet P Schaper W Clauss M VEGFR-1-selective VEGF homologue PlGF is arteriogenic: evidence for a monocyte-mediated mechanism Circ Res 2003 92 378 385 12600898 10.1161/01.RES.0000057997.77714.72

[B18] Murakami M Zheng Y Hirashima M Suda T Morita Y Ooehara J Ema H Fong GH Shibuya M VEGFR1 tyrosine kinase signaling promotes lymphangiogenesis as well as angiogenesis indirectly via macrophage recruitment Arterioscler Thromb Vasc Biol 2008 28 658 664 18174461 10.1161/ATVBAHA.107.150433

[B19] Fantin A Vieira JM Gestri G Denti L Schwarz Q Prykhozhij S Peri F Wilson SW Ruhrberg C Tissue macrophages act as cellular chaperones for vascular anastomosis downstream of VEGF-mediated endothelial tip cell induction Blood 2010 116 829 840 20404134 10.1182/blood-2009-12-257832 PMC2938310

[B20] Care AS Diener KR Jasper MJ Brown HM Ingman WV Robertson SA Macrophages regulate corpus luteum development during embryo implantation in mice J Clin Invest 2013 123 3472 3487 23867505 10.1172/JCI60561 PMC3726148

[B21] Ferrara N Gerber HP LeCouter J The biology of VEGF and its receptors Nat Med 2003 9 669 676 12778165 10.1038/nm0603-669

[B22] Shibuya M Vascular endothelial growth factor (VEGF) and its receptor (VEGFR) signaling in angiogenesis: a crucial target for anti- and Pro-angiogenic therapies Genes Cancer 2011 2 1097 1105 22866201 10.1177/1947601911423031 PMC3411125

[B23] Shibuya M Vascular endothelial growth factor receptor-1 (VEGFR-1/Flt-1): a dual regulator for angiogenesis Angiogenesis 2006 9 225 230 discussion 231 17109193 10.1007/s10456-006-9055-8

[B24] Fong GH Rossant J Gertsenstein M Breitman ML Role of the Flt-1 receptor tyrosine kinase in regulating the assembly of vascular endothelium Nature 1995 376 66 70 7596436 10.1038/376066a0

[B25] Hiratsuka S Minowa O Kuno J Noda T Shibuya M Flt-1 lacking the tyrosine kinase domain is sufficient for normal development and angiogenesis in mice Proc Natl Acad Sci 1998 95 9349 9354 9689083 10.1073/pnas.95.16.9349 PMC21341

[B26] Luttun A Tjwa M Moons L Wu Y Angelillo-Scherrer A Liao F Nagy JA Hooper A Priller J De Klerck B Compernolle V Daci E Bohlen P Dewerchin M Herbert JM Fava R Matthys P Carmeliet G Collen D Dvorak HF Hicklin DJ Carmeliet P Revascularization of ischemic tissues by PlGF treatment, and inhibition of tumor angiogenesis, arthritis and atherosclerosis by anti-Flt1 Nat Med 2002 8 831 840 12091877 10.1038/nm731

[B27] Wu Y Zhong Z Huber J Bassi R Finnerty B Corcoran E Li H Navarro E Balderes P Jimenez X Koo H Mangalampalli VR Ludwig DL Tonra JR Hicklin DJ Anti-vascular endothelial growth factor receptor-1 antagonist antibody as a therapeutic agent for cancer Clin Cancer Res 2006 12 6573 6584 17085673 10.1158/1078-0432.CCR-06-0831

[B28] Vorontchikhina MA Zimmermann RC Shawber CJ Tang H Kitajewski J Unique patterns of Notch1, Notch4 and Jagged1 expression in ovarian vessels during folliculogenesis and corpus luteum formation Gene Expr Patterns 2005 5 701 709 15939383 10.1016/j.modgep.2005.02.001

[B29] Kim M Park HJ Seol JW Jang JY Cho YS Kim KR Choi Y Lydon JP Demayo FJ Shibuya M Ferrara N Sung HK Nagy A Alitalo K Koh GY VEGF-A regulated by progesterone governs uterine angiogenesis and vascular remodelling during pregnancy EMBO Mol Med 2013 5 1415 1430 23853117 10.1002/emmm.201302618 PMC3799495

[B30] Brachtendorf G Kuhn A Samulowitz U Knorr R Gustafsson E Potocnik AJ Fassler R Vestweber D Early expression of endomucin on endothelium of the mouse embryo and on putative hematopoietic clusters in the dorsal aorta Dev Dyn 2001 222 410 419 11747076 10.1002/dvdy.1199

[B31] Nakhuda GS Zimmermann RC Bohlen P Liao F Sauer MV Kitajewski J Inhibition of the vascular endothelial cell (VE)-specific adhesion molecule VE-cadherin blocks gonadotropin-dependent folliculogenesis and corpus luteum formation and angiogenesis Endocrinology 2005 146 1053 1059 15591148 10.1210/en.2004-0977

[B32] Groten T Fraser HM Duncan WC Konrad R Kreienberg R Wulff C Cell junctional proteins in the human corpus luteum: changes during the normal cycle and after HCG treatment Hum Reprod 2006 21 3096 3102 16923746 10.1093/humrep/del286

[B33] Rosmarin AG Weil SC Rosner GL Griffin JD Arnaout MA Tenen DG Differential expression of CD11b/CD18 (Mo1) and myeloperoxidase genes during myeloid differentiation Blood 1989 73 131 136 2562920

[B34] Keenihan SN Robertson SA Diversity in phenotype and steroid hormone dependence in dendritic cells and macrophages in the mouse uterus Biol Reprod 2004 70 1562 1572 14766730 10.1095/biolreprod.103.024794

[B35] De M Choudhuri R Wood GW Determination of the number and distribution of macrophages, lymphocytes, and granulocytes in the mouse uterus from mating through implantation J Leukoc Biol 1991 50 252 262 1856596 10.1002/jlb.50.3.252

[B36] De M Wood GW Analysis of the number and distribution of macrophages, lymphocytes, and granulocytes in the mouse uterus from implantation through parturition J Leukoc Biol 1991 50 381 392 1833492 10.1002/jlb.50.4.381

[B37] Brandon JM Macrophage distribution in decidual tissue from early implantation to the periparturient period in mice as defined by the macrophage differentiation antigens F4/80, macrosialin and the type 3 complement receptor J Reprod Fertil 1995 103 9 16 7707305 10.1530/jrf.0.1030009

[B38] Paria BC Zhao X Das SK Dey SK Yoshinaga K Zonula occludens-1 and E-cadherin are coordinately expressed in the mouse uterus with the initiation of implantation and decidualization Dev Biol 1999 208 488 501 10191061 10.1006/dbio.1999.9206

[B39] Chakraborty I Das SK Dey SK Differential expression of vascular endothelial growth factor and its receptor mRNAs in the mouse uterus around the time of implantation J Endocrinol 1995 147 339 352 7490564 10.1677/joe.0.1470339

[B40] Wang ES Teruya-Feldstein J Wu Y Zhu Z Hicklin DJ Moore MA Targeting autocrine and paracrine VEGF receptor pathways inhibits human lymphoma xenografts in vivo Blood 2004 104 2893 2902 15238424 10.1182/blood-2004-01-0226

[B41] Huang H Shen J Vinores SA Blockade of VEGFR1 and 2 suppresses pathological angiogenesis and vascular leakage in the eye PLoS One 2011 6 e21411 21731737 10.1371/journal.pone.0021411 PMC3120882

[B42] Carmeliet P Moons L Luttun A Vincenti V Compernolle V De Mol M Wu Y Bono F Devy L Beck H Scholz D Acker T DiPalma T Dewerchin M Noel A Stalmans I Barra A Blacher S VandenDriessche T Ponten A Eriksson U Plate KH Foidart JM Schaper W Charnock-Jones DS Hicklin DJ Herbert JM Collen D Persico MG Synergism between vascular endothelial growth factor and placental growth factor contributes to angiogenesis and plasma extravasation in pathological conditions Nat Med 2001 7 575 583 11329059 10.1038/87904

[B43] Fraser HM Duncan WC SRB reproduction, fertility and development award lecture 2008. Regulation and manipulation of angiogenesis in the ovary and endometrium Reprod Fertil Dev 2009 21 377 392 19261215 10.1071/rd08272

[B44] De M Wood GW Influence of oestrogen and progesterone on macrophage distribution in the mouse uterus J Endocrinol 1990 126 417 424 2212933 10.1677/joe.0.1260417

[B45] Kirsch TM Friedman AC Vogel RL Flickinger GL Macrophages in corpora lutea of mice: characterization and effects on steroid secretion Biol Reprod 1981 25 629 638 7030418 10.1095/biolreprod25.3.629

